# Therapeutic effect of human adipose-derived stem cells and their secretome in experimental diabetic pain

**DOI:** 10.1038/s41598-017-09487-5

**Published:** 2017-08-29

**Authors:** Anna T. Brini, Giada Amodeo, Lorena M. Ferreira, Anna Milani, Stefania Niada, Giorgia Moschetti, Silvia Franchi, Elisa Borsani, Luigi F. Rodella, Alberto E. Panerai, Paola Sacerdote

**Affiliations:** 1 Department of Biomedical, Surgical and Dental Sciences, University of Milan, via Vanvitelli 32, 20129 Milan, Italy; 2 IRCCS Galeazzi Orthopaedic Institute, via Galeazzi 4, 20161 Milan, Italy; 3 Dipartimento di Scienze Farmacologiche e Biomolecolari, Universita’ degli Studi di Milano, via Vanvitelli 32, 20129 Milan, Italy; 4 Division of Anatomy and Physiopathology, Department of Clinical and Experimental Sciences, University of Brescia, viale Europa 11, 25123 Brescia, Italy

## Abstract

Painful neuropathy is one of the complications of diabetes mellitus that adversely affects patients’quality of life. Pharmacological treatments are not fully satisfactory, and novel approaches needed. In a preclinical mouse model of diabetes the effect of both human mesenchymal stromal cells from adipose tissue (hASC) and their conditioned medium (hASC-CM) was evaluated. Diabetes was induced by streptozotocin. After neuropathic hypersensitivity was established, mice were intravenously injected with either 1 × 10^6^ hASC or with CM derived from 2 × 10^6^ hASC. Both hASC and CM (secretome) reversed mechanical, thermal allodynia and thermal hyperalgesia, with a rapid and long lasting effect, maintained up to 12 weeks after treatments. In nerves, dorsal root ganglia and spinal cord of neuropathic mice we determined high IL-1β, IL-6 and TNF-α and low IL-10 levels. Both treatments restored a correct pro/antinflammatory cytokine balance and prevented skin innervation loss. In spleens of streptozotocin-mice, both hASC and hASC-CM re-established Th1/Th2 balance that was shifted to Th1 during diabetes. Blood glucose levels were unaffected although diabetic animals regained weight, and kidney morphology was recovered by treatments. Our data show that hASC and hASC-CM treatments may be promising approaches for diabetic neuropathic pain, and suggest that cell effect is likely mediated by their secretome.

## Introduction

Diabetes mellitus (DM) is one of the most common and serious chronic diseases around the world^1^ and diabetic peripheral neuropathy is one of the most frequent complications of DM. Sixty-six percent of people with Type 1 DM and 59% of people with Type 2 DM have objective evidence of peripheral neuropathy^2^. The pathophysiology of diabetic neuropathy (DN) remains complex and not fully elucidated and it has multipathogenic mechanisms that cause a diversity of physical symptoms: allodynia, hyperalgesia, numbness and cutaneous ulceration^3^. Persistent neuropathic pain interferes significantly with quality of life, impairing sleep, and emotional well-being, and it is a significant causative factor for anxiety, loss of sleep, and non-compliance with treatment. Unfortunately, most of the available analgesic drugs are not satisfactory in controlling diabetic neuropathic pain,for both insufficient efficacy and side effects^1, 2^.

Recently, we and others have demonstrated that pro- and anti-inflammatory cytokines, produced by immune cells as well as by glia and microglia in nerve, dorsal root ganglia and spinal cord, are involved in neuropathic pain^4–6^. They start a cascade of neuroinflammation-related events that may maintain and worsen the original injury, participating in pain generation and chronicization^4–7^. A large activation of inflammatory cascade, proinflammatory cytokine upregulation, and neuroimmune communication pathways plays a vital role in structural and functional damage of peripheral nerves, leading to the diabetic peripheral neuropathy^8, 9^.

Mesenchymal stem/stromal cells (MSCs) and in particular adipose-derived stromal cells, known to have therapeutic potential and likely translational advantages^10, 11^, may offer a novel therapeutic option to treat DN. MSCs modulate the nervous system injured environment and promote repair as they secrete anti-inflammatory and anti-apoptotic molecules, and trophic factors to support axonal growth, immunomodulation, angiogenesis, remyelination, and protection from apoptotic cell death^11^. Transplanted MSCs not only directly differentiate into neurons and endothelial cells, but also secrete a broad range of biologically active factors and extracellular vesicles (EVs), generally referred as MSC secretome. Secretome analysis demonstrates that it contains elevated concentrations of FGF, VEGF-A, and nerve growth factor which are involved in nerve and vascular tissue health. Furthermore, ASCs are known to be immunomodulatory through the regulation of immune cells by mechanisms which include both direct cellular contact and release of soluble factors such as TGF-β, IL-10, leukemia inhibitory factor (LIF) and others^12^. It has been shown that ASCs reduce allogeneic lymphocytes response by displaying potent immunosuppressive effects and that MSCs suppress both effectors T cell and inflammatory responses^12, 13^.

We previously demonstrated that human ASC treatment reduced both allodynia and hyperalgesia and normalized neuroinflammation in a murine model of neuropathy induced by sciatic nerve chronic constriction injury (CCI)^14^.

Despite the fact that MSCs were initially proposed for cell therapy based on their differentiation potential, the lack of correlation between functional improvement and cell engraftment or differentiation at the site of injury has led to suggest that MSCs exert their effects primarily through their secreted products and not only through their differentiation potential. Many studies provide pivotal support for this paracrine hypothesis and MSC therapy is increasingly rationalized on MSC secreted factors rather than on their differentiation ability^15^.

Here we analyze the therapeutic effect of hASC-conditioned media (secretome, hASC-CM) and compare it with cell treatment in a streptozotocin mouse model of Type 1-diabetes. Animal behavior was evaluated through mechanical or cold allodynia and thermal hyperalgesia, together with the profile of the main pro and antinflammatory cytokines involved in nociception transmission at the main neural sites relevant for pain. Cytokines produced by peripheral lymphocyte are also studied. We also evaluated the effect of hASC-CM and hASC on skin thickness and innervation and on renal injury, evident in this preclinical model^16, 17^.

We demonstrate that just a single intravenous injection of either hASC or hASC-CM irreversibly reverts the established neuropathic hypersensitivity, blunts neuroinflammation, restores skin innervation and reduces peripheral immune activation. Furthermore, the induced nephropathy is counteracted by both treatments, providing evidence for kidney damage protection, too.

## Results

### In STZ-diabetic mice hASC-conditioned medium and hASC treatments exert a fast and long lasting relief of sensory hypersensitivity

**Figure 1:**
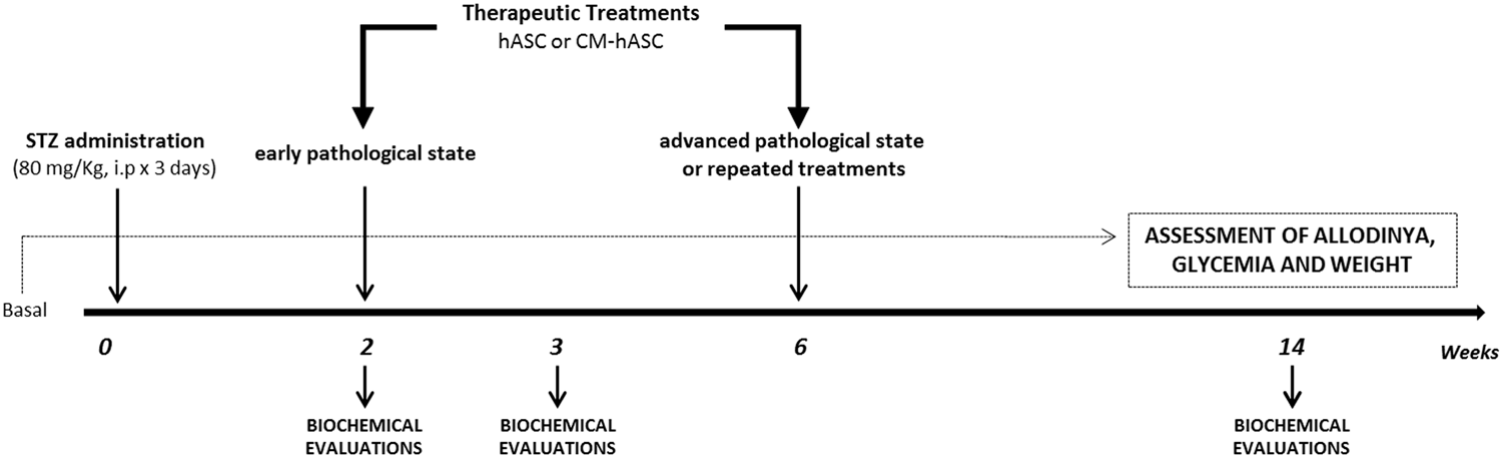
Experimental design. STZ: streptozotocin; hASC: human adipose-derived stem/stromal cells (1 × 10^6^ cells). hASC-CM: hASC-conditioned medium (from 2 × 10^6^ cells).

A scheme of the experimental protocols of the study is described in Fig. 1.

**Figure 2:**
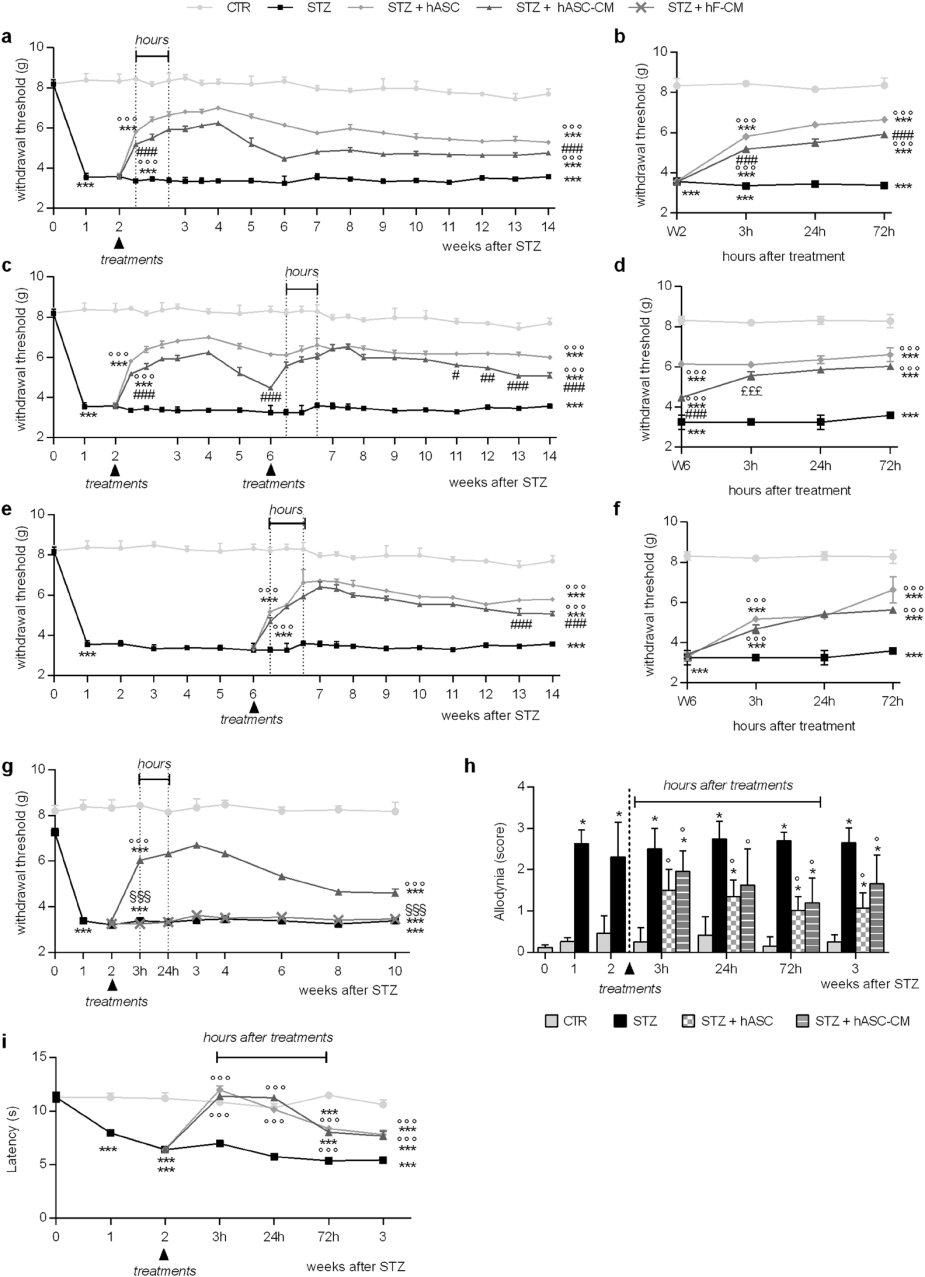
hASC and hASC-CM treatments reduce allodynia in STZ mice. (**a**–**f**) Effects of i.v. hASC (1 × 10^6^) or hASC-CM (obtained from 2 × 10^6^ cells) treatments on mechanical allodynia in STZ mice. (**a**,**b**) Mice received a single hASC or hASC-CM injection, 2 weeks after STZ, and the effects were monitored up to 14 weeks after STZ (long-lasting effects) (**a**) or few hours (3 to 72 h) after the injection (short-term effects) (**b**). (**c,d**) Mice received two repeated hASC or hASC-CM administrations 2 and 6 weeks after STZ. Long-lasting effects (**c**) and short-term effects after the second administration (**d**). (**e,f**) Mice received a single hASC or hASC-CM injection, 6 weeks after STZ. Long-lasting effects (**e**) and short-term effects (**f**). (**g**) Effect of hASC-CM and CM obtained from human fibroblasts (hF-CM), administered 2 weeks after STZ. Data represent mean ± SEM of 6–8 mice per group. Two-way ANOVA followed by Bonferroni’s test was used for multiple comparisons. ***p < 0.001 vs CTR; °°°p < 0.001 vs STZ; ^#^p < 0.05, ^##^p < 0.01, ^###^p < 0.001 vs STZ + hASC; ^§§§^p < 0.001 vs STZ + hASC-CM; ^£££^p < 0.001 vs. W6. (**h**,**i**) effect of hASC and hASC-CM on thermal allodynia (**h**) and hyperalgesia (**i**). Treatments were performed 2 weeks after STZ and cold allodynia and hot plate thesholds were evaluated after 3, 24, 72 hours and 1 week after treatments. Values are mean ± SEM of 6 mice per group, and were compared with Mann–Whitney U-test (cold allodynia) and two-way ANOVA followed by Bonferroni’s test for multiple comparisons (thermal hyperalgesia). *p < 0.05, ***p < 0.001 vs CTR; °p < 0.05, °°°p < 0.001 vs STZ.

One week after STZ the paw withdrawal thresholds (PWT) of diabetic mice were reduced compared to control and they were maintained significantly lower up to 14 weeks (Fig. 2, panel a, two way ANOVA, p < 0.001 vs CTR).

Two weeks after STZ, when mechanical allodynia was fully developed, hASC or hASC-CM were i.v. injected. As reported in Fig. 2, panel a, both treatments were able to significantly reduce mechanical allodynia (two way ANOVA, p < 0.001 vs STZ), although the effect of hASC was significantly stronger than that elicited by hASC-CM (two way ANOVA, p < 0.001). A significant reduction of mechanical allodynia was evident already 3 hours after treatments (Fig. 2, panel b, p < 0.001). Their anti-allodynic effect was maximal at 2 weeks and was extremely long lasting since allodynia was maintained significantly reduced up to 12 weeks after a single hASC and hASC-CM injection (Panel a, p < 0.001 vs STZ).

Moreover, 4 weeks after the first treatment (6 weeks after STZ) a group of diabetic animals was administered a second time with either hASC or hASC-CM. As shown in Fig. 2 panel c, the second hASC-CM injection increased the PWT restoring sensitivity to the level reached after the first treatment and prolonging its effect. The anti-allodynic effect was potentiated few hours after the second hASC-CM injection (Fig. 2, panel d; PWT 3 hours after second injection vs. PWT before second injection, p < 0.001) and its trend completely mimicked both the rapid effect evoked by the early treatment (compare panels b and d) and the hASC effect up to 10 weeks after diabetes- induction (panel c). The repeated hASC treatment did not further ameliorate allodynia.

In order to elucidate whether hASC and hASC-CM treatments were also therapeutically effective at a later time point, animals were treated 6 weeks after diabetes induction. Also this late treatment was able to provide a fast and irreversible antiallodynic effect (Fig. 2, panels e and f, p < 0.001 vs STZ). In addition, 13 weeks after STZ, the effect of hASC appeared significantly more pronounced than the one exerted by CM (p < 0.001 hASC vs.hASC-CM).

To provide evidence that hASC-CM effect was due to the specificity of the cell source we treated STZ-mice with conditioned media derived from human fibroblasts (hF-CM). As shown in Fig. 2 panel g, hF-CM does not counteract mechanical allodynia, indicating that specific factors contained in the secretome of adipose-derived mesenchymal stem/stromal cells are responsible of the effect observed *in vivo*.

Since the sensory alterations associated to diabetes neuropathy in patients are often diverse and associated to modified response to several stimuli, we also decided to test the action of hASC and hASC-CM treatments on cold allodynia and thermal hyperalgesia during the first week after injection. As shown in Fig. 2 panel h, the cold allodynia displayed by STZ-diabetic animals was significantly reduced by both treatments (p < 0.05 vs STZ). Similarly, as reported in panel i, administration of both hASC and hASC-CM rapidly reverted thermal hyperalgesia, and their effect was still present 1 week later (p < 0.001 vs STZ), demonstrating the ability of hASC and their conditioned media to relieve hypersensitivity to different stimuli that are peculiar of diabetic pain.

### Localization of hASC

**Figure 3:**
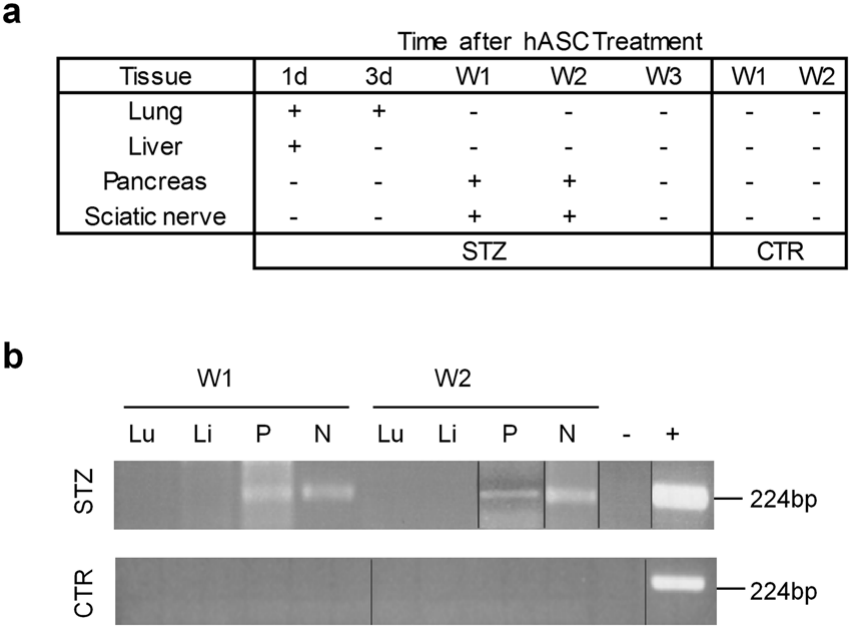
hASC tracking by human ALU sequence detection. Summary of the Alu PCR products observed in lung, liver, pancreas and sciatic nerve after 1 and 3 days and 1, 2 and 3 weeks from hASC administration (**a**). Representative gels showing human ALU sequence detection in STZ and naïve (CTR) mice tissues collected 1 week (W1) and 2 weeks (W2) after treatment (**b**). Lu: lung; Li: liver; P: pancreas; N: sciatic nerve; + : positive control; −: negative control.

We monitored the fate of the injected hASC, and investigated the localization of infused hASC up to 3 weeks after injection. The presence of human DNA was assessed in lungs, liver, pancreas and sciatic nerve of both diabetic (STZ) and healthy (CTR) mice (Fig. 3, panels a and b). As expected, at day 1 after treatment, human DNA was present in filter organs such as lungs and liver, at 3 days only in lung and it was not detectable in the damaged tissues such as pancreas and nerve (Fig. 3 panel b, and data not shown).

However, when the antiallodynic effect was well established (week 1 and week 2 after injection), human DNA was noticeable only in pancreas and sciatic nerve of STZ-mice, confirming the tropism and recruitment of hASC to the injured tissues, and not to the CTR-undamaged ones (Fig. 3, panels a and b). At later time, 21 days after injection, human DNA was undetectable in all the tested tissues (Fig. 2, panel b and data not shown).

### hASC-CM and hASC treatments restore cytokine levels in sciatic nerve, Dorsal Root Ganglia (DRG) and spinal cord

It is well known that in diabetic neuropathy a neuroinflammatory cascade, characterized by altered levels of pro- and anti inflammatory cytokines, is present in the main sites involved in nociception transmission. To verify an anti-inflammatory or immunomodulatory mechanism for hASC- and hASC-CM mediated anti-hypersensitivity action, we checked IL-1β, IL-6, TNFα and IL-10 levels in the sciatic nerve, DRG and spinal cord of STZ mice.

**Figure 4:**
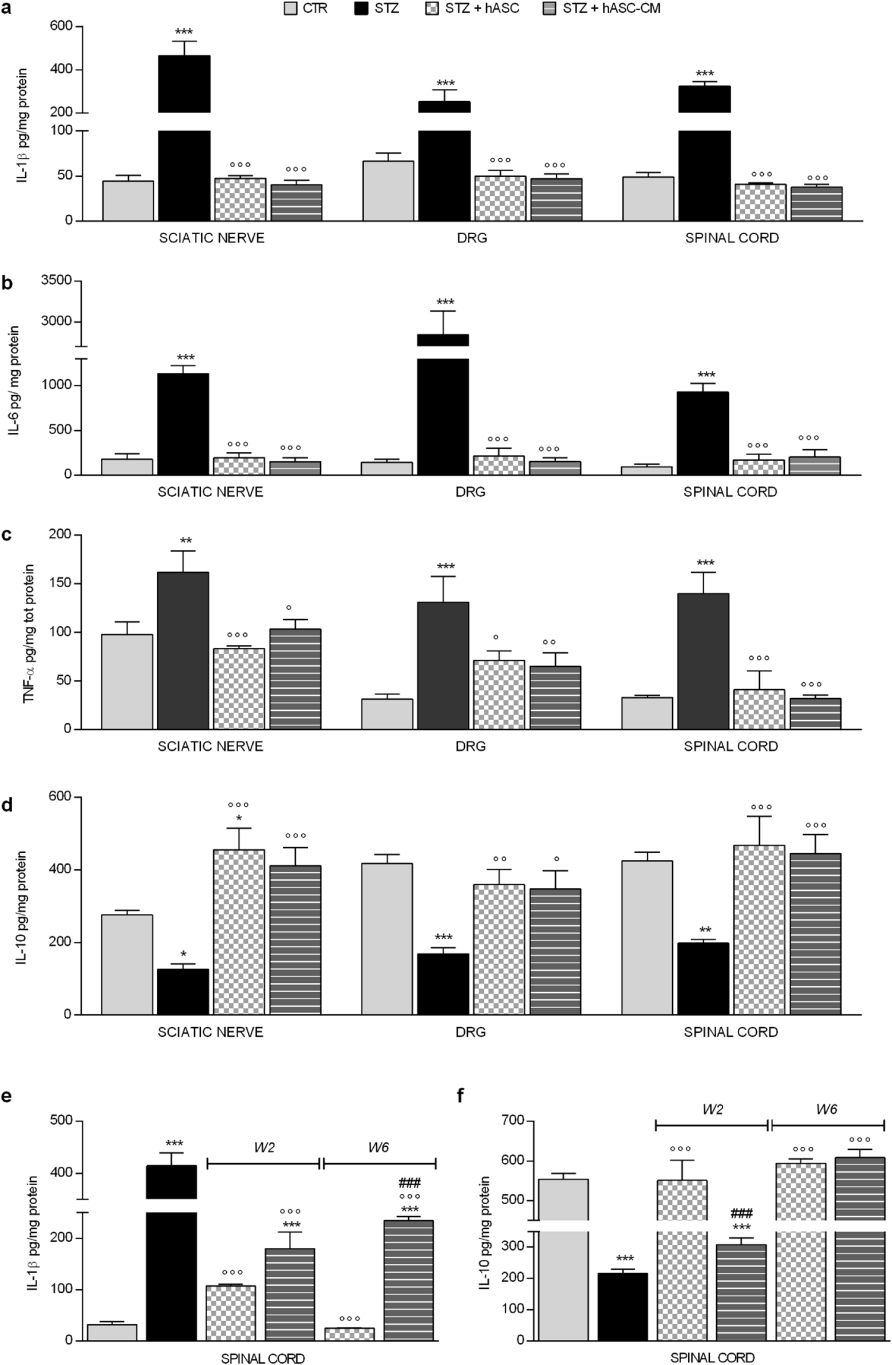
hASC and hASC-CM maintain a correct pro- and anti-inflammatory cytokine balance in sciatic nerves, DRG and spinal cord of STZ mice. IL-1β, IL-6, TNFα and IL-10 protein content in nervous tissues was evaluated by ELISA and reported as pg cytokine/mg total protein. (**a**–**d**) IL-1β (**a**) IL-6 (**b**), TNF-α (**c**) and IL-10 (**d**) in sciatic nerve, DRG and spinal cord of STZ mice treated 2 weeks after STZ with hASC or hASC-CM; cytokines were evaluated after 1 week from treatments. (**e**,**f**) IL-1β (**e**) and IL-10 (**f**) levels in spinal cord, measured 14 weeks after STZ in animals treated with hASC or hASC-CM either 2 weeks (W2) or 6 weeks (W6) after STZ. Data represent mean ± SEM of 6 mice per group. One-way ANOVA was used for statistical evaluation, followed by Bonferroni’s post hoc test for multiple comparisons. *p < 0.05, **p < 0.01, ***p < 0.001 vs CTR; °p < 0.05, °°p < 0.01, °°°p < 0.001 vs STZ; ^###^p < 0.001 vs STZ + hASC.

Panels a–d of Fig. 4 illustrate cytokine levels 3 weeks from neuropathy induction and 1 week after hASC and hASC-CM injection. Proinflammatory cytokines IL-1β, IL-6 and TNF-α (Fig. 4, panel a–c) were overexpressed in the peripheral (sciatic nerve and DRG) and central (spinal cord) nervous system of diabetic mice (p < 0.01 vs non diabetic mice). Both hASC and hASC-CM treatments were able to restore IL-1β, IL-6 and TNF-α basal levels, 1 week after treatment (p < 0.05 vs diabetic animals). In addition, IL-10 levels (Fig. 4 panel d) appeared significantly reduced in diabetic animals in sciatic nerve (p < 0.05), DRG (p < 0.001) and spinal cord (p < 0.01), and both hASC and hASC-CM significantly increased IL-10 concentrations in DRG (p < 0.05) and spinal cord (p < 0.001). In the sciatic nerve of treated animals the antinflammatory cytokine was significantly elevated in comparison with STZ animals with both treatments (p < 0.001). Indeed after hASC, IL-10 increased over basal levels (p < 0.05), indicating a rapid switch towards an antinflammatory environment in all these areas involved in pain transmission.

Fourteen weeks after STZ, spinal cord IL-1β levels were still significantly elevated (p < 0.001) and IL-10 levels were reduced (p < 0.001) in diabetic, indicating the persistence of neuroinflammation, as shown in panels e and f of Fig. 4. As previously described for the antiallodynic effect (Fig. 2), the modulation of cytokines induced by hASC and hASC-CM was long lasting. Twelve weeks after treatments (W2), the levels of IL-1β were still significantly reduced by hASC and hASC-CM (p < 0.001, panel e), while only in animals treated with hASC we observed a significant normalization of IL-10 (p < 0.001, panel f).

Both treatments were still effective when administered at a later stage of the pathology (W6 after STZ); as reported in Fig. 4 panels e and f, 8 weeks after administration both hASC and their CM significantly modulated cytokine levels (p < 0.001), although the effect on IL-1β was more evident with hASC than with hASC-CM treatment (p < 0.001 hASC vs hASC-CM).

Finally, to investigate a rapid effect on cytokine modulation, we measured IL-1β and IL-10 levels in sciatic nerve, DRG and spinal cord 2 weeks from STZ, 3 hours after hASC and hASC-CM treatment. The results are reported in Supplementary Figure S1. Two weeks after STZ, IL-1β levels were significantly elevated and IL-10 levels decreased in all nervous system. The acute treatments were already able to positively modulate cytokine levels, since they were not any more different from CTR, although a complete restoration was evident only for IL-1β in sciatic nerve and IL-10 in DRG (p < 0.05vs. STZ, Supplementary Figure S2).

### Effect of hASC and hASC-CM on DRG and spinal cord Calcitonin Gene Related Petide(CGRP) levels

**Figure 5:**
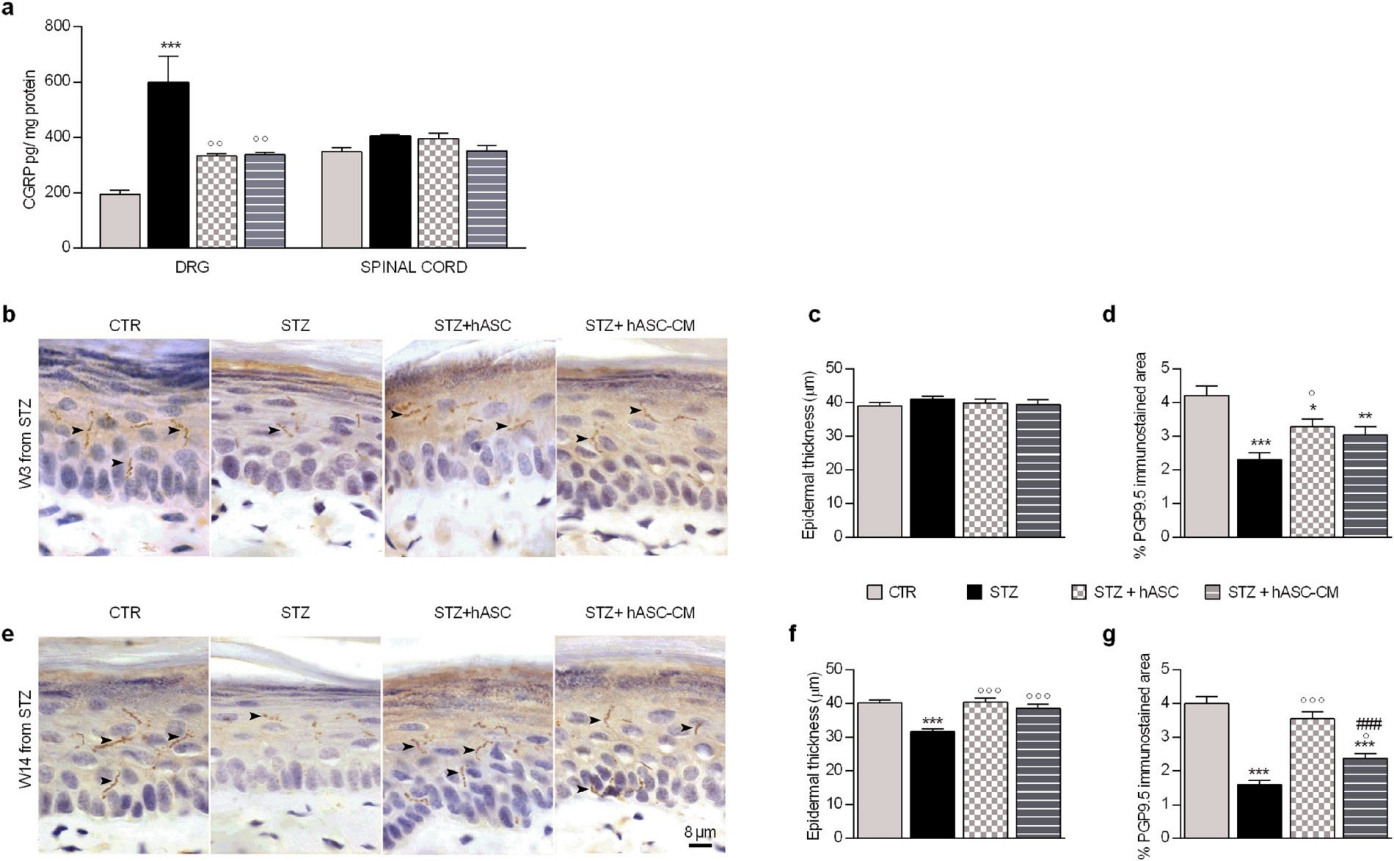
hASC and hASC-CM prevent alteration of DRG CGRP, thickness reduction and nerve fiber loss in paw skin of STZ mice. (**a**) DRG and spinal cord levels of CGRP measured 3 weeks after STZ in diabetic mice treated with hASC or hASC-CM 2 weeks after STZ. Data represent mean ± SEM of 6 mice per group. One-way ANOVA was used for statistical evaluation, followed by Bonferroni’s post hoc test for multiple comparisons. ***p < 0.001 vs CTR; °°p < 0.01 vs STZ. Microphotographs of plantar skin after PGP9.5 + immunohistochemistry and counterstaining with haematoxylin at 3 (**b**) and 14 weeks (**e**) after STZ, in CTR, STZ, STZ + hASC and STZ + hASC-CM groups. Animals were treated 2 weeks after STZ. Nerve fibers are stained in brown; arrow heads indicate PGP9.5^+^ fibers. Quantitative evaluation of epidermal thickness at 3 weeks (**c**) and 14 weeks (**f**) and of PGP9.5 immunopositivity as percentage of immunopositive area in epidermal and subepidermal area at 3 (**d**) and 14 (**g**) weeks after STZ. Data represent mean ± SEM and were compared by One-way ANOVA followed by a Bonferroni’s multiple comparison test. *p < 0.05, **p < 0.01, ***p < 0.001 vs CTR; °p < 0.05, °°°p < 0.001 vs STZ; ^###^p < 0.001 vs STZ + hASC.

In order to further confirm the effect of treatments on hypersensitivity, we measured the effect of h-ASC and hASC-CM on CGRP protein levels in DRG and spinal cord of STZ mice. CGRP levels were significantly elevated in DRG of STZ mice 3 weeks after diabetes induction, and both treatments were able to significantly re-establish them one week after administration (Fig. 5, panel a). We did not find any CGRP modification in spinal cord of diabetic mice (Fig. 5, panel a).

### hASC and hASC-conditioned media normalize skin thickness and PGP9.5^+^nervous fibers

Neuropathic pain is associated with epidermal thinning and reduced innervation, measured as expression of Protein Gene Product 9.5 (PGP 9.5) in axons of the epidermis and dermis^17^. Both skin thickness and cutaneous innervation of the plantar skin were evaluated 3 and 14 weeks after STZ diabetic induction, corresponding to 1 and 12 weeks after hASC and hASC-CM treatment (Fig. 5 panels b–g). Three weeks after STZ, skin thickness was not yet altered in the STZ mice and neither treatments had any effect (Fig. 5, panels b and c). At fourteen weeks, STZ animals showed a significant decrease of skin thickness (Fig. 5, panels e and f, p < 0.001). Both hASC and hASC-CM prevented this decrease since the skin thickness of treated mice was significantly greater than diabetic mice one (p < 0.001; Fig. 5, panels e and f).

In addition, the high density of PGP9.5^+^ nervous fibers distributed both in epidermis and in subepidermal layer in CTR mice is progressively decreased in STZ mice. The PGP9.5^+^ nervous fibers were significantly decreased 3 (panels b and d) and 14 (panels e and g) weeks after STZ (p < 0.001). One week after hASC treatment, neuropathic animals showed a density of PGP9.5^+^ fibers significantly higher respect to the STZ group (p < 0.05) but still lesser respect to the CTR (p < 0.05; Fig. 5, panel d). No significant effect of hASC-CMs on density was observed. Fourteen weeks after STZ (12 weeks from treatments, panel g) hASC-treated animals recovered the density of PGP9.5^+^ nerve fibers to the same level of the CTR group (p < 0.001 vs STZ). In hASC-CM-treated animals, a significantly higher density of PGP9.5^+^ nervous fibers was present, especially at subepithelial levels, respect to STZ group (p < 0.05), although it never reached the CTR values (Fig. 5, panels e and g).

### Effect of hASC-conditioned media and hASC on weight loss, hyperglycemia,and glomerulopathy in STZ-mice

**Figure 6:**
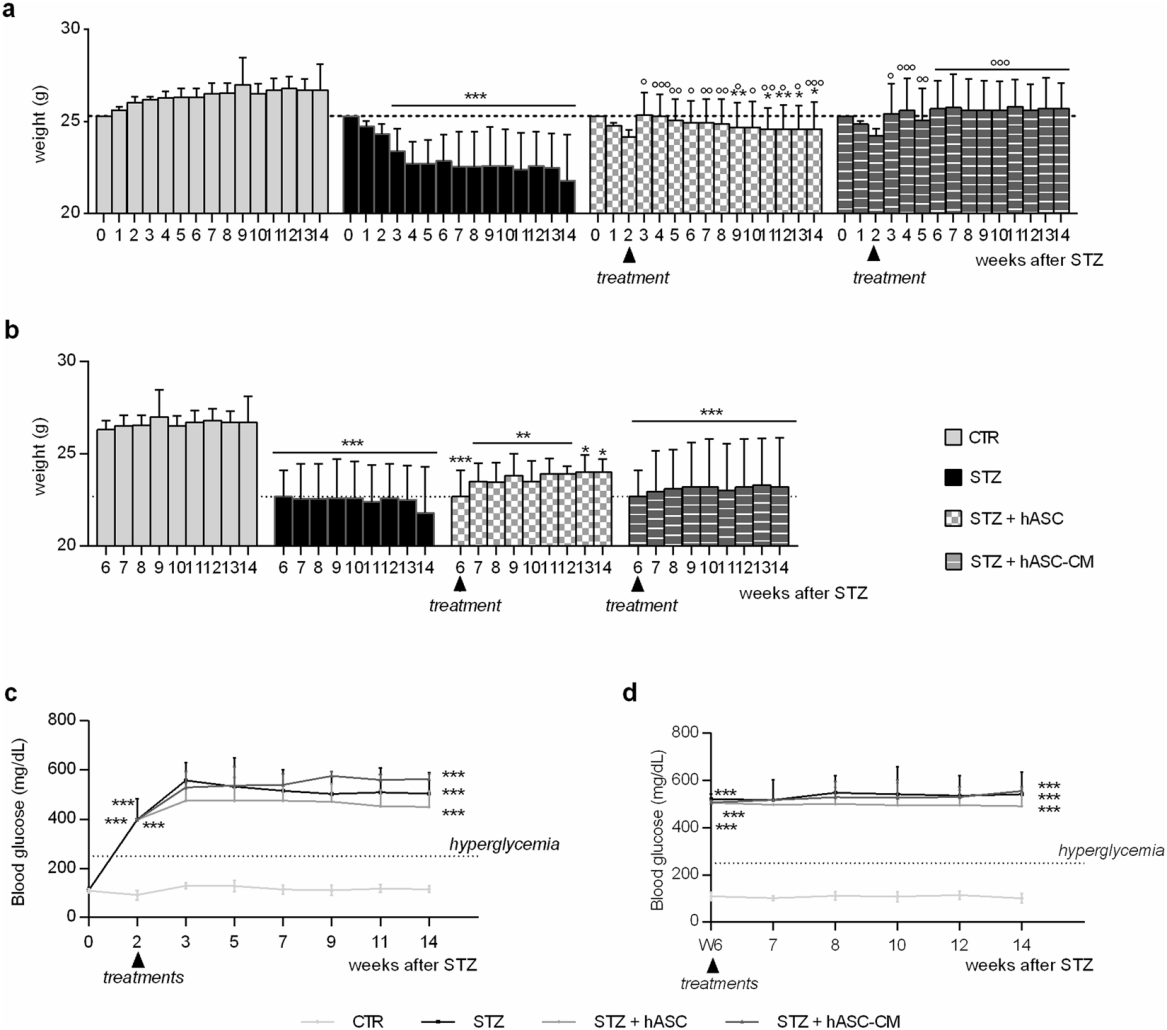
Effect of hASC and hASC-CM on body weight and blood glucose levels in STZ mice. (**a,b**) Body weight of STZ mice treated with hASC or hASC-CM 2 weeks (**a**) and 6 weeks (**b**) after STZ. (**c**,**d**) Blood glucose levels of STZ mice treated with hASC or hASC-CM 2 weeks (**c**) and 6 weeks (**d**) after STZ. Data are means ± SD of 6/8 animals. Two-way ANOVA was used for statistical evaluation, followed by Bonferroni’s test for multiple comparisons. *p < 0.05, **p < 0.01, ***p < 0.001 vs CTR; °p < 0.05, °°p < 0.01, °°°p < 0.001 vs STZ.

Three weeks after diabetic induction body weight significantly decreased in STZ mice (Fig. 6, panel a, p < 0.001). Surprisingly, both the hASC and hASC-CM administration significantly prevented the loss of body weight (p < 0.05, Fig. 6, panel a), despite the fact that neither hASC nor hASC-CMs were able to revert the body weight loss if administered 6 weeks after diabetes induction, as shown in panel b. In addition, no treatments modified blood glucose levels that were always elevated in STZ mice over time (Fig. 6, panels c and d).

Since renal injury is a well known diabetes complication, we also focused our attention on glomerulopathy, which is a primary evidence of type I diabetes nephropathy. Analysing hematoxylin/eosin kidney sections, an evident expansion of Bowman’s space of about 114% was observed in diabetic mice compared to CTR animals, and both hASC and hASC-CM treatments restored it (Supplementary Figure S2).

### hASC and hASC-CM modulate splenocyte cytokine production

The STZ multiple low-doses protocol used in our study is known to develop an autoimmune response against pancreatic tissue which is sustained by a Th1 pattern of activation^18, 19^. For this reason we decided to investigate whether a T-helper polarization was present in splenocytes from diabetic mice and whether hASC or hASC-CM did exert any immunomodulatory activity.

**Figure 7:**
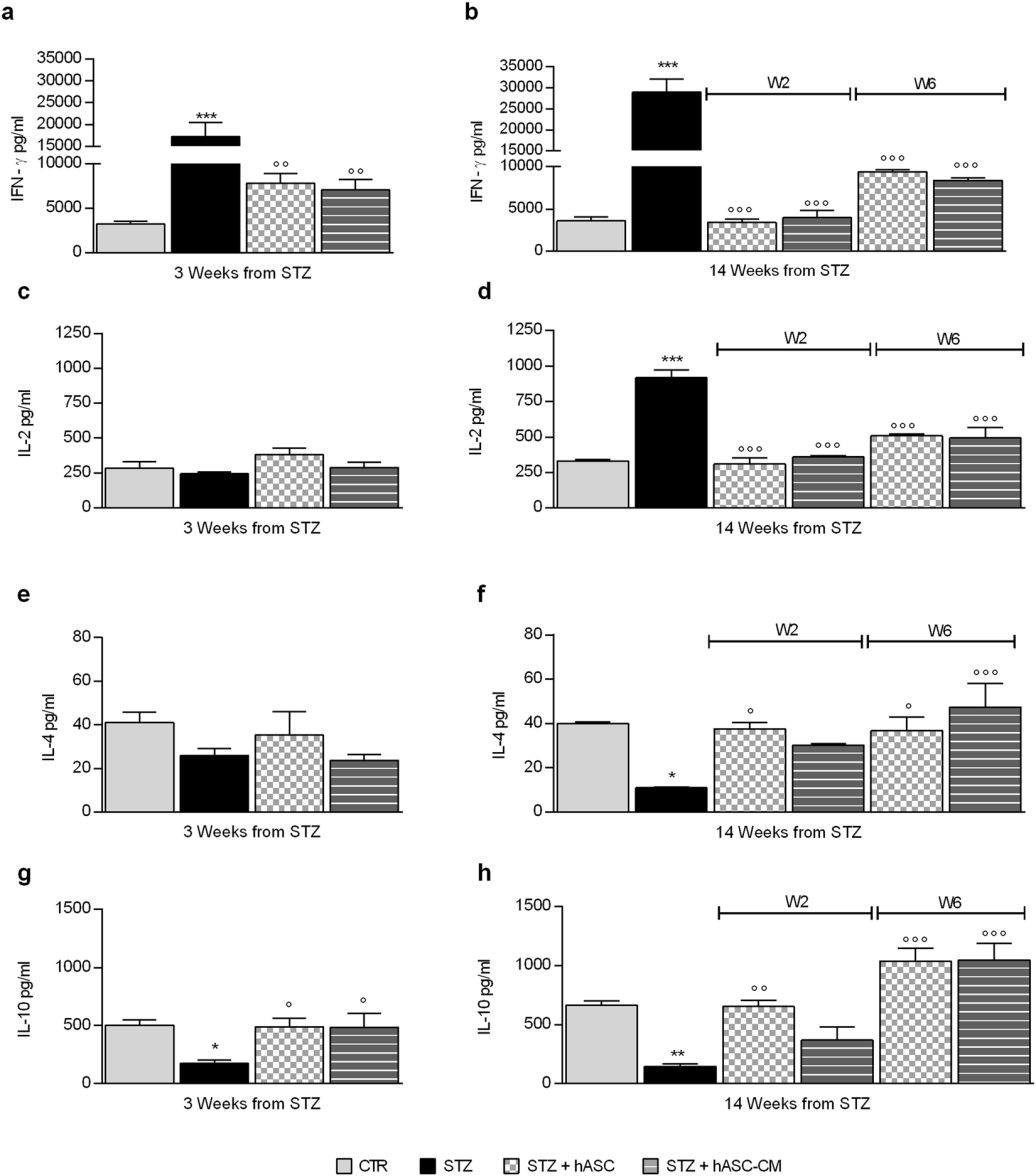
hASC and hASC-CM treatments modulate cytokine release from splenocytes. IFN-γ (**a** and **b**), IL-2 (**c** and **d**), IL-4 (**e** and **f**) and IL-10 (**g** and **h**) were evaluated by ELISA, and reported as protein concentrations in culture media. (**a**,**c**,**e** and **g**) depict the levels of cytokine evaluated 3 weeks after STZ and 1 week after hASC or hASC-CM treatments. (**b**,**d**,**f** and **h**) Report cytokines levels measured 14 weeks after STZ in animals treated with hASC or hASC-CM either 2 weeks (W2) or 6 weeks (W6) after STZ. Data represent mean ± SEM of 6 mice per group, and have been statistically analyzed with One-way ANOVA, followed by Bonferroni’s test for multiple comparisons. *p < 0.05, **p < 0.01, ***p < 0.001 vs CTR; °p < 0.05, °°p < 0.01, °°°p < 0.001 vs STZ.

As reported in panels a, c, e and g of Fig. 7, 3 weeks after STZ, ConA-stimulated splenocytes released higher levels of IFN-γ (panel a, p < 0.001 vs. CTR), while IL-10 release (panel g) was reduced (p < 0.05 vs CTR). In one week, hASC and hASC-CM treatments were able to re-establish both IFN-γ (p < 0.01 vs STZ) and IL-10 (p < 0.05 vs STZ) normal levels (Fig. 7, panels a and g, respectively).

When cytokine levels were measured 14 weeks after diabetes induction, a clear shift toward a Th1 pattern was present, characterized by higher IFN-γ and IL-2 secretion (Fig. 7, panels b and d, respectively, p < 0.001) and lower IL-4 (panels f, p < 0.05) and IL-10 levels (panel h, p < 0.01). hASC and hASC-CM treatments were able to normalize cytokine levels, as demonstrated by the IFN-γ and IL-2 decrease (panels b and d, p < 0.001 vs STZ) and IL-10 and IL-4 increase when treatments were performed 6 weeks after diabetes induction and cytokines measured 8 weeks later (Fig. 7, panels f and h). When the effect was evaluated 12 weeks after administration, both hASC and hASC-CM were able to restore IFN-γ and IL-2 (Fig. 7, panels b and d, p < 0.001 vs STZ). IL-4 and IL-10 reached basal levels only in hASC-treated mice (IL-4 p < 0.05 and IL-10 p < 0.01 vs STZ) while in hASC-CM-treated animals the recovery was not complete (Fig. 7, panels f and h), suggesting that the effects exerted by hASC is more long lasting than the hASC-CM one. Altogether, our data indicate that both hASC and hASC-CM treatments are able to counteract the Th1 polarization developed in this experimental diabetes model.

## Discussion

Diabetic neuropathic pain is characterized by sensory alterations, including hypoaesthesia, tactile and thermal allodynia, and current treatments are often active on only some of them^20^. Our results demonstrate that hASC and their secretome can control diabetic complications such as neuropathic hypersensitivity, acting on several peripheral and central mechanisms involved in the development and maintenance of this condition, such as neural and immune elements.

Systemic treatments with either hASC or hASC-CM are able to significantly relieve tactile and cold allodynia as well as heat hyperalgesia, although the effect of cells remains significantly higher than CM one. The concept that MSC’s secretome may be responsible for the beneficial effects of stem cell therapy is now a prevalent theory^13, 21, 22^. However,this is the first report demonstrating that the conditioned medium of hASC also results in quite similar therapeutic effects in diabetic neuropathic condition and comparing simultaneously the effect of CM and of the stem cells from which it has been produced.

One of the most striking result of our study is related to the time course of the antiallodynic effect exerted by both hASC and hASC-CM. In fact, their antiallodynic effect was not only rapid but also extremely long lasting, an effect that is hardly reached by any analgesics clinically used^23, 24^.

Furthermore, the long lasting antiallodynic effect of hASC-CM, up to 14 weeks, may lead to speculate that a direct engrafment of stem cells in the nervous tissue has to be excluded, favouring the hypothesis of a precocious reprogramming of the immune and neuronal environments that, once activated, changes the course of the neuropathy. In a juxtacrine or paracrine fashion hASC can crosstalk and modulate endogenous stem cells that may also induce nerve regeneration, as previously described in a different model of neuropathy^25^.

Similarly, the biologically active substances contained in the hASC-CM can activate/deactivate specific signaling pathways with a final protective outcome. It must be underlined that CM derived from human dermal fibroblasts are completely inactive on allodynia, confirming that the mesenchymal stem feature is a fundamental pre-requisite in this novel approach.

The ability of hASC and their secretome to modulate the host cell response is demonstrated by the effect on the cytokine levels in the nervous tissue exerted by both treatments. IL-1β, IL-6, TNF-a and IL-10 in the peripheral (nerves, DRG) and central (spinal cord) nervous system are now recognized as a pivotal signal for maintenance of neuropathic pain, regardless of its origin^4, 26, 27^. Here we demonstrated that one week after hASC and hASC-CM treatment, neuroinflammation is significantly blunted and a correct balance between IL-1β, IL-6, TNF-a and IL-10 is re-established. Interestingly, the levels of IL-10 after treatments in the sciatic nerves are even more elevated than those measured in normal animals, confirming the relevant role for this cytokine in controlling sensory hypersensitivity. In parallel with the long lasting modulation of allodynia, also the effect of hASC on IL-1β and IL-10 in spinal cord is fully maintained up to 12 weeks after treatment, suggesting that once the switch toward an antinflammatory program has been started, it is permanently sustained. Interestingly, at later times after administration, the effect of hASC-CM on IL-10 is not as evident as the one of stem cells themselves. Moreover, we also demonstrate that the modulation of cytokine levels begins almost immediately after hASC or hASC-CM, since 3 hours after treatment we already observed a trend to restoration of IL-1β/10 balance in nervous tissues, (Supplementary Figure S1), that becomes fully significant 1 week after treaments.

Since clinical diabetic neuropathy is a progressive disorder, and its manifestation may need several years to develop^27^, it was important to assess whether hASC and hASC-CM were able to modify allodynia and neuroinflammation also when administered at a later stage of the disease. In our model, hyperglycemia and abnormal sensitivity are developed soon after STZ injection, while longer time is necessary for structural nerve modifications^28^. Interestingly both hASC and hASC-CM were fully effective in reverting allodynia and restoring IL-1β, /IL-10 spinal cord balance also when injected 6 weeks after diabetes induction. This result is particularly relevant since, although a tight control of glucose in the patients may successfully slow the progression of NP, no established curable treatment is available during the progressive stage^24^. Quantifying the density of intraepidermal nerve fiber to assess cutaneous innervation is considered a reliable mean of both diagnosing and staging diabetic neuropathy^17, 29, 30^. We decided to measure PGP-9.5 expression, since as shown in prior studies^31–34^, anti-PGP 9.5 labels all known types of peripheral innervation. Here we showed that a nerve fiber damage began in 3 weeks and was gradually aggravated in untreated diabetic group. These results are in agreement with previous findings in diabetic rats^35^. We observed that both treatments were able to ameliorate the loss of nerve fiber density detected by PGP9.5^+^ nerve fiber, further supporting a neuroprotective activity in diabetes.We are however aware that further immunohistochemical analysis with specific fibers’markers would be important to assess which fibers are lost and eventually recovered by treatments.

This significant effect on diabetic pain is also corraborated by the restoration of CGRP levels that we observe in DRG of diabetic mice, further demonstrating that both hASC and hASC-CM are able to modulate several important mediators or neurotransmitters involved in pain sensitivity.

MSCs carry out pleiotropic effects on the immune system by both secreting bioactive molecules and by cell-cell contact involving dendritic cells, B and T cells^36, 37^. A possible explanation of the efficacy of hASC and hASC-CM in alleviating allodynia may be an immediate modulation of peripheral immune responses. Inflammation and immune activation have been recognized as fundamental mechanisms in the pathophysiology of diabetes and of its complications^38^. An autoimmune reactivity characterized by a T helper 1 profile is consistently present in clinical diabetes. We find that a progressive polarization of peripheral immune response towards a Th1 profile is present in our STZ model and we demonstrate that hASC are involved in modulating the peripheral immune response. In fact, hASC treatment reduced IL-2 and IFN-γ release by peripheral splenocytes and increased IL-10 and IL-4 secretion, keeping an optimal Th1/Th2 balance. Moreover, this effect is almost completely mimicked also by hASC-CM, indicating that cell-cell contact is not absolutely necessary for immune modulation, that appears to be mainly due to a paracrine mode of action.

Based on these results we can envisage that hASC, when i.v. injected in diabetic animals, may start to release bioactive factors that immediately modulate allodynia, immune responses and thereafter convert a pro-inflammatory/neuro-destructive environment to an antinflammatory/neuroprotective one. We showed that hASC are recruited at the site of lesioned tissue, such as pancreas and nerves of diabetic mice, while they are undetectable when the cells are i.v. injected in naïve animals. Furthermore, we suggest that hASC disappear after reprogramming the tissue cells, since cellular life span in the lesioned tissue (2 weeks) is shorter than the duration of their effects (12 weeks), as already reported^39^. In the case of hASC-CM, the cocktail of bioactive mediators may exert a similar precocious modulation of host tissue, that is maintained over-time. However, at longer time after the single injection, the hASC positive effect on the antinflammatory cytokines appears stronger, suggesting that either the recruitment of cells at the injured sites or the cell to cell contact with endogenous immune cells may be useful. The positive long lasting effect of hASC-CM have been already described in different models of pathological conditions, such as liver disease, urological disfunction and Alzheimer^40–42^, and here we demonstrated that a repeated injection of hASC-CM promotes a higher antiallodynic response and further extends its duration, giving promising indications for a future clinical treatment.

At the moment, we cannot exclude that the neuroprotective effects of hASC and hASC-CM might also be mediated by an improvement of neural vascularity that is induced by the angiogenic factors contained in hASC secretome. Nonetheless, this aspect, previously suggested by others^43, 44^, can be combined with the effects described here of the hASC and their CM.

The content and the relevance of hASC-CM factors is still under study. Our CM obtained from hASC maintained in similar culturing conditions as previously described by others^13, 21, 45, 46^, should contain a wide range of cytokines, chemokines, and growth factors such as BDNF, VEGF, IGF. Recently a paper by Chen *et al*.^47^, suggested that the release of TGF-β by bone marrow stromal cells may be particularly involved in the modulation of neuropathic pain in the CCI and spare nerve injury models. From preliminary analysis conducted, we know that also our hASC-CM contain high levels of TGF-β (Sacerdote and Brini, unpublished observation), that may play an important role also in diabetes neuropathic pain.

We also detected the presence of exosomes in hASC-CM (Brini, unpublished observations), and the secreted exosomes carry specific mRNA or miRNA, which could potentiate the reparative process and heal the injured tissues^22, 48^. Although it is possible that for different pathological conditions, diverse mediators may be responsible for the beneficial effect, we think that what makes the secretome unique is just the simultaneous presence of all these multiple factors. To confirm our hypothesis, we have also shown the hASC-CM effect on another diabetes alteration such as renal injury. This diabetes complication is due to the progressive inflammation and immune activation promoted by the formation of advanced glycation-end products, oxidative stress, and activation of renin-angiotensin-aldosterone system within the kidney^49, 50^. Indeed, inflammation activated by the metabolic, biochemical and haemodynamic derangements plays a key role in the development and progression of diabetic nephropathy^51^. Since in STZ-mice kidney damage is ameliorated by both treatments, we hypothesize that hASC and hASC-CM may control several aspects of diabetes linked to inflammation and immune activation. Weight loss is also a classical symptom associated to the STZ diabetes models, and a general positive effect of hASC and hASC-CM treatments is demonstrated by the prevention of weight loss in treated mice^28, 38^. Several factors may contribute to the prevention of weight loss that we observed in treated mice. In STZ mice, altered protein and lipid catabolism due either to hyperglycemia, to the inflammatory condition and oxidative stress are likely at the basis of weight loss^28^. Moreover, it has been suggested that the presence of allodynia and painful symptoms may affect the locomotor activity and modify energy expenditure^52^. Interestingly, as in our work, a reduction of body weight loss has been observed also in the absence of modification of the hyperglycemic state in STZ rats^52^. It can be suggested that the reduction of systemic inflammation and hypersensitivity may have a role. It can also be hypothesized that hASC and hASC- CM may contribute with hormones and mediators directly involved in the control of adipogenesis and body weight^53^. This hypothesis, however, deserves to be studied in future work.

No significant change in blood glucose levels was observed, suggesting that the efficacious relief of nociceptive hypersensitivity is independent of glycemia. The use of mesenchymal stem cells of several origin, including hASC, has been reported to induce beta cell protection in the STZ models^19, 54–57^. We indeed observed the presence of hASC in lesioned pancreas starting from day 7 after administration. However, this discrepancy can rely on the different administration route, systemically or intra-pancreatic resulting in different numbers of cells reaching the pancreas, and, most of all, the timing of administration. In our study in fact, hASC and hASC-CM were injected 2 weeks after STZ, when hyperglycemia was developed and pancreas already damaged.

In the near future, we plan to extend our experiments with other routes of administration of either hASC or their CM. In particular results obtained after intraplantar and intratechal route of administration will be compared to the present ones after i.v administration, in order to better understand the peripheral vs. central sites of action.

Both here and in our previous study^14^ we never observed any significant variability among the *in vivo* effect of the different lots of hASCs and their conditioned medium used in all the treatments. However to overcome the different cells growth of the, in the future we might consider to set a more standardized production of cells and CM pooling several ASC populations, as also suggested by Bodle *et al*.^58^.

In conclusion, we confirm and explain the ability of i.v. hASC to exert a long lasting control of diabetic neuropathic hypersensitivity showing, for the first time, that the effect of the hASC is mimicked by their secretome, confirming the general view that stem cells act mainly throughout a paracrine action.

The safety of autologous MSCs has been documented by a number of clinical trials^10, 59, 60^ and the use of secretome could be further safer. In addition, considering all the studies on the effects of MSCs on diabetes, and knowing that peripheral neuropathy affects up to 60% of diabetic patients^2^, we believe that advanced diabetic neuropathy could become a first clinical target for this type of medicine cellular product. Thinking about the future, a novel therapeutic option with hASC secretome might be suggested for treating advanced peripheral painful neuropathy.

## Materials and Methods

### Isolation and culture of human Adipose-derived Stem/Stromal Cells (hASC) and human dermal fibroblasts (hDFs)

All methods involving human specimens were performed following relevant guidelines and regulations. Human tissues were waste materials from abdominoplasty and liposuction performed at IRCCS Galeazzi Orthopaedic Institute.We followed the procedure PQ 7.5.125, version 4, 22.01.2015, approved by IRCCS Galeazzi Orthopaedic Institute, regarding waste materials to be used for research purpose. As required by the procedure mentioned above, written informed consent was obtained from all patients and all the samples were anonymized. Adipose-derived stem/stromal cells were isolated from subcutaneous adipose tissue of 6 healthy donors (1 male and 5 females, age range 26–53 y/o), as previously described^61^. Adipose tissue samples were digested with 0.75 mg/ml type I Collagenase (250 U/mg, Worthington Biochemical Corporation); stromal vascular fraction was filtered and deriving cells were cultured (10^5^ cells/cm^2^; 37 °C, 5% CO_2_) in control medium (cDMEM: DMEM supplemented with 10% FBS - ThermoFisher Scientific Hyclone-, 2 mM L-glutamine, 50 U/ml penicillin, 50 µg/ml streptomycin). Upon reaching 70–80% confluence, cells were detached with 0.5% trypsin/0.2% EDTA and expanded (reagents, when not otherwise indicated, were provided by Sigma-Aldrich). Characterized hASCs^10, 61^ were administered between the third and seventh-passage.

Human dermal fibroblasts (hDFs) were obtained from de-epidermized dermis of one healthy donor (female, 26 y/o). The tissue fragments were digested with 0.1% collagenase type I at 37 °C for 6 hours then centrifuged and the pellet resuspended in cDMEM and seeded for the expansion.

### Conditioned media production

Upon reaching 80–90% confluence, cells were washed and cultured in serum-free DMEM (phenol-free DMEM supplemented with 2 mM L-glutamine, 50 U/ml penicillin, 50 µg/ml streptomycin) for 72 hours. Conditioned media were then centrifuged at 800 g for 10 minutes to remove cell debris, and concentrated by a factor of about 45 times using Amicon^®^ Ultra-15 centrifugal filter columns with 3-kDa molecular weight cutoff (Millipore). The volume of CM is indicated respect to the cell number.

### Animals and *in vivo* study design

All animal care and experimental procedures complied with the International Association for the Study of Pain and European Community (E.C.L358/118/12/86) guidelines and were approved by the Animal Care and Use Committee of the Italian Ministry of Health (Permission 21/2014 to AP and 470/2016 to PS). All efforts were made to minimize animal suffering and to reduce the number of animals used. Studies involving animals are reported in accordance with the ARRIVE guidelines for reporting experiments involving animals^62^. A total of 120 animals were used in the experiments described here. Each experiment consisted of 6–8 mice/group (see Statistic for details). C57BL/6 J male mice weighing 20–25 g, 9 weeks old (Envigo, Italy) were housed with light/dark cycles of 12 hours, temperature of 22 ± 2 °C, humidity of 55 ± 10%, food and water *ad libitum*.

Upon receipt, animals were randomized in cages of 3 mice each. After 1 week cages were randomly allocated to the different experimental groups (diabetes or vehicle controls).

Diabetes was induced by intraperitoneal (i.p.) administration of Moderate Low Doses of streptozotocin (STZ) (80 mg/kg daily for three consecutive days)^8^, (Sigma Aldrich, Italy), in citrate buffer 0.1 M, pH 4.55. Control mice were injected with citrate buffer.

Tail-vein blood glucose concentration was assessed using a glucometer (GLUCOCARD G + meter, Menarini diagnostics, Italy). Animals with glucose values above 250 mg/dl were considered diabetic. Animal weight was monitored every week.

### Experimental protocol

Experimental protocol is depicted in Fig. 1. Diabetic mice were randomly allocated to different groups of treatment. Two weeks after STZ treatment (W2), either CM derived from 2 × 10^6^ hASC (hASC-CM) or 1 × 10^6^ hASC resuspended in 200 µl of PBS supplemented with 2.5% heparin, were injected intravenously through the caudal vein. Control animals were administered with PBS + 2,5% heparin only^14, 63^.

Moreover, 6 weeks after STZ (W6) two groups of mice received a second hASC or hASC-CM treatment (4 weeks after the first treatment), and further STZ-groups were treated for the first time at this advanced stage of disease.

Mechanical allodynia was evaluated before diabetes induction, 1 and 2 weeks after streptozotocin administration, 3, 24 and 72 hours after either hASC-CM or hASC treatment, and every 7 days up to 14 weeks. Cold allodynia and thermal hyperalgesia were determined at 3, 24 and 72 hours and 1 week after treatments.

Biochemical analyses were performed at different time points from hASC/hASC-CM treatment.

Animals were sacrificed: 3 hours after hASC/hASC-CM treatment (2 weeks from STZ); 3 weeks after STZ, corresponding to 1 week after hASC/hASC-CM treatment; 14 weeks after STZ corresponding to either 12 or 8 weeks after hASC-CM or hASC treatment.

### Mechanical Allodynia

Mechanical allodynia was tested evaluating the mechanical touch sensitivity with a blunt probe on the mid plantar surface of the hind paw, using the Dynamic Plantar Aesthesiometer (Ugo Basile, Italy)^10, 17^. Responses to mechanical stimuli, (paw withdrawal thresholds, PWT) were measured before neuropathy induction (0), and after STZ on both hind paws by researchers who were blind to treatments.

### Cold allodynia

Cold allodynia was evaluated as previously described^64^. Briefly, a drop (50 µl) of acetone was placed in the middle of the plantar surface of the hind paw. The mouse behaviour was monitored during the first 20 s. If the mouse did not withdraw, flick or stamp the hindpaw within this 20 s period, then no response was recorded (0). However if within this 20 s period the animal responded to the cooling effect of the acetone, then its response was assessed for an additional 20 s. Responses to acetone were graded according to the following 4 points scale: 0, no response; 1, quick withdrawal, flick or stamp of the paw; 2, prolonged withdrawal or repeated flicking (more than twice) of the paw; 3, repeated flicking of the paw with licking directed at the plantar surface of the hind paw. Acetone was applied alternately 3 times to each hind paw and the responses were scored. Mean scores were then generated for each mouse. Researchers were blind to treatments.

### Thermal hyperalgesia

The hot-plate test was used to assess thermal hyperalgesia. The apparatus was set at a temperature of 54 ± 0.5 °C. Each animal was placed on the heated surface, and the time interval (seconds, s) between placement and the simultaneous licking of both fore paws was recorded. The cut-off time, chosen to avoid tissue damage, was 30 s.

### Tissue collection and storage

Mice were killed by CO_2_ inhalation for spinal cord (L4-L6), dorsal root ganglia (L4-L6), sciatic nerves, kidney, lung, liver, pancreas, plantar skin and spleen dissection. Tissues were either immediately frozen in liquid nitrogen and stored at −80 °C until use or fixed for histological analysis.

### Measurement of cytokines and CGRP level in nervous tissues

For cytokine extraction, spinal cord, dorsal root ganglia (DRG) and sciatic nerves samples were homogenized in lysis buffer (ice-cold PBS + protease inhibitor cocktail, Roche Diagnostics, Italy). Tissues were centrifuged at 1000 g for 15 min at 4 °C and supernatants used to measure cytokines levels and total protein content (Lowry’s method). For CGRP extraction, DRG and spinal cords were homogenized in 2 N acetic acid, heat at 90 °C for 10 minutes, centrifuged, dried and the supernatant dissolved in EIA assay buffer (Cayman Chemical, SpiBio, Italy).

### Splenocyte collection and *in vitro* Stimulation for Cytokine Assay

Splenocytes were adjusted in 24-well plates at the final concentration of 4 × 10^6^ cells/ml of culture medium (RPMI 1640 with 10% FCS, 1% glutamine, 2% antibiotics and 0.1% 2-mercaptoethanol) and incubated at 37 °C in 5% CO_2_ and 95% air with 10 μg/ml Concanavalin A (ConA) for T helper (Th)1 and Th2 cytokine stimulation. After 24 (for IFN-γ and IL-2) or 48 hours (for IL-4 and IL-10) of culture, times of maximum release^8, 65^, supernatants were stored at −80 °C.

### ELISA

Cytokine concentration was determined using ultra-sensitive ELISA kits according to the manufacturer’s instruction. DuoSet ELISA development systems for mouse IL-2, IFN-γ and IL-4 were from R&D Systems (Minneapolis, USA) while mouse IL-1β, IL-6, TNFα and IL-10 ELISA Ready-SET-Go from eBioscience (San Diego, CA). Cytokine concentrations were reported as pg cytokine/mg total protein content in sciatic nerve, DRG and spinal cord. Cytokine production by splenocytes was reported as concentrations in media of stimulated cultures.

CGRP was assayed with mouse/rat CGRP Enzyme Immunoassay Kit (EIA, Cayman Chemical, SpiBio, Italy) and data reported as pg/mg total protein content.

### Skin Immunohistochemistry

The plantar skin was collected from 3 mice/group, and fixed in 10% buffered formalin for 24 hours. The skin was embedded in paraffin according to standard procedures and cut at 8 μm by a microtome (Microm HM 325). Alternate paraffin sections from the middle part of plantar skin were evaluated by immunohistochemistry. The sections were deparaffinised, rehydrated and subjected to antigen retrieval in 0.05 M sodium citrate buffer (pH 6.0) in hot water bath (98 °C for 20′). Endogenous peroxidase activity was blocked by incubation with 3% hydrogen peroxide. Sections were immunostained with the monoclonal antibody anti-mouse PGP9.5 (protein gene product 9.5, dilution 1:250, EMD Millipore, Darmstadt, Germany), a marker of nerve fibers. All sections were processed using UltraVisionQuanto Detection System horseradish Peroxidase (HRP; ThermoScientific, Bio-Optica, Milan, Italy), followed by development with diaminobenzidine (Amresco, Prodotti Gianni, Milan, Italy). Finally, the sections were dehydrated and mounted and some of them was previously counterstained with hematoxylin. The immunohistochemical control was performed by omitting the primary antibody, in presence of isotype matched IgGs and performing pre-adsorption using the related peptide. Digitally fixed images of slices were analyzed using an image analyzer (Image Pro-Premier, Immagini e Computer, Milan, Italy). The density of nerve fibers was evaluated measuring the percent area occupied by positive PGP9.5 nerve fibers in epidermal area including the sub-basal one. The data of all animals were analyzed at a final 400X magnification measuring three randomly collected fields for each section by researchers unaware of the animal group assignment.

### Morphological evaluation of epidermal thickness

Digitally fixed images of slices were analyzed using an image analyzer (Image Pro-Premier, Immagini e Computer, Milan, Italy). The epidermal thickness was evaluated by means of the distance (μm) from basal membrane to stratum granulosum at a final 400X magnification measuring three randomly collected fields for each section by researcher unaware of the animal group assignment.

### Kidney histological analysis

Kidneys were fixed in 10% buffered formalin (Sigma-Aldrich) and then processed for paraffin embedding. Morphological analysis was performed on 2–3 μm Harris’ hematoxylin and eosin (Bio Optica) stained sections (images acquired by Olympus BX51, Japan). Bowman’s space area^66^ in at least five randomly collected fields were quantified by ImageJ software at a final 200X magnification, by researchers unaware of the animal group assignment. At least 30 glomeruli/group were analyzed.

### hASC localization by Alu sequence detection

Genomic DNA from lungs, livers, pancreas and sciatic nerves of STZ and control mice was collected at day 1, 3, 7, 14 and 21 after 1 × 10^6^ hASC i.v. injection. Briefly, 25 mg of each tissue (except for the sciatic nerve) were homogenized and lysed in 0.5 ml of lysis buffer (1% SDS, 400 mM NaCl, 5 mM EDTA [pH 8.0], 100 mM Tris [pH 8.0]) containing 0.2 mg/ml of Proteinase K (Sigma-Aldrich). Differently, both sciatic nerves from each mouse were lysed in 50 µl of lysis buffer. Samples were incubated overnight at 56 °C and following phenol/chloroform extraction, DNA was precipitated in ethanol and resuspended in MilliQ water. Primate specific ALU sequences were amplified by PCR using appropriate primers: (forward, 5′-TGGGCGACAGAACGAGATTCTAT-3′; reverse, 5′-CTCACTACTTGGTGACAGGTTCA-3′) that produce DNA amplicons of 224 bp. Human DNA isolated from hASC was used as positive control.

### Statistic

Statistical analysis was performed using GraphPad Prism 5 Software (San Diego, CA, U.S.A). Data were tested for equal variance before choosing statistical analysis.

Data from mechanical allodynia measurements and thermal hyperalgesia were analyzed by mean of two way ANOVA considering the type of treatment and the time as factors. Baseline values, i.e. responses before STZ injection, were not included in the analysis. If an overall test comparing group was significant, Bonferroni’s test was used for between-group comparisons in the post hoc analysis. Cold allodynia scores were compared with Mann–Whitney U-test.

Cytokines and CGRP results were analyzed using one-way Anova, followed by Bonferroni’s post hoc test for multiple comparison. Body weight and blood glucose levels were statistically evaluated by Two-way ANOVA considering the type of treatment and the time as factors. If an overall test comparing group was significant, Bonferroni’s test was used for between-group comparisons in the post hoc analysis.

Data from histological experiments (skin thickness and kidney damage) and of immunohistochemistry (PGP9.5) were analyzed and compared by one-way ANOVA and by a Bonferroni’s multiple comparison test.

The overall significance level was 0.05 for each hypothesis. Each group consisted of 6 animals. The group size was chosen on the basis of the results obtained in our previous studies. For antiallodynic response, considering an expected difference in means of 40%, SD of 10%, number of treatments 4, power 0.95 and α 0.05, a number of 3 animals for group could have been enough. However, considering also cytokines as important outcome, where the SD can be estimated in 30%, the number must be increased to 6 animals.

Results are expressed as mean ± SD or SEM.

## Electronic supplementary material

supplementary information
