# Supplementary material for: Therapeutic effect of human adipose-derived stem cells and their secretome in experimental diabetic pain
Source: Sci Rep. 2017 Aug 29;7:9904. doi: 10.1038/s41598-017-09487-5 (PMC5575274; doi:10.1038/s41598-017-09487-5)
Supplement: Supplementary file 1 — supplementary information [file 41598_2017_9487_MOESM1_ESM.pdf]

Therapeutic effect of human adipose-derived stem cells and their secretome in experimental diabetic pain

Anna T Brini[a,b], Giada Amodeo[c], Lorena M. Ferreira[a,b], Anna Milani[a,b], Stefania Niada[a,b], Giorgia Moschetti[c], Silvia Franchi[c], Elisa Borsani[d], Luigi F. Rodella[d], Alberto E Panerai[c], Paola Sacerdote[c]*

[a]Department of Biomedical, Surgical and Dental Sciences, University of Milan, via Vanvitelli 32, 20129 Milan, Italy

[b]IRCCS Galeazzi Orthopaedic Institute, via Galeazzi 4, 20161 Milan, Italy

[c]Dipartimento di Scienze Farmacologiche e Biomolecolari, Universita' degli Studi di Milano, via Vanvitelli 32, 20129 Milan, Italy

[d]Division of Anatomy and Physiopathology, Department of Clinical and Experimental Sciences, University of Brescia, viale Europa 11, 25123 Brescia, Italy

Supplementary data

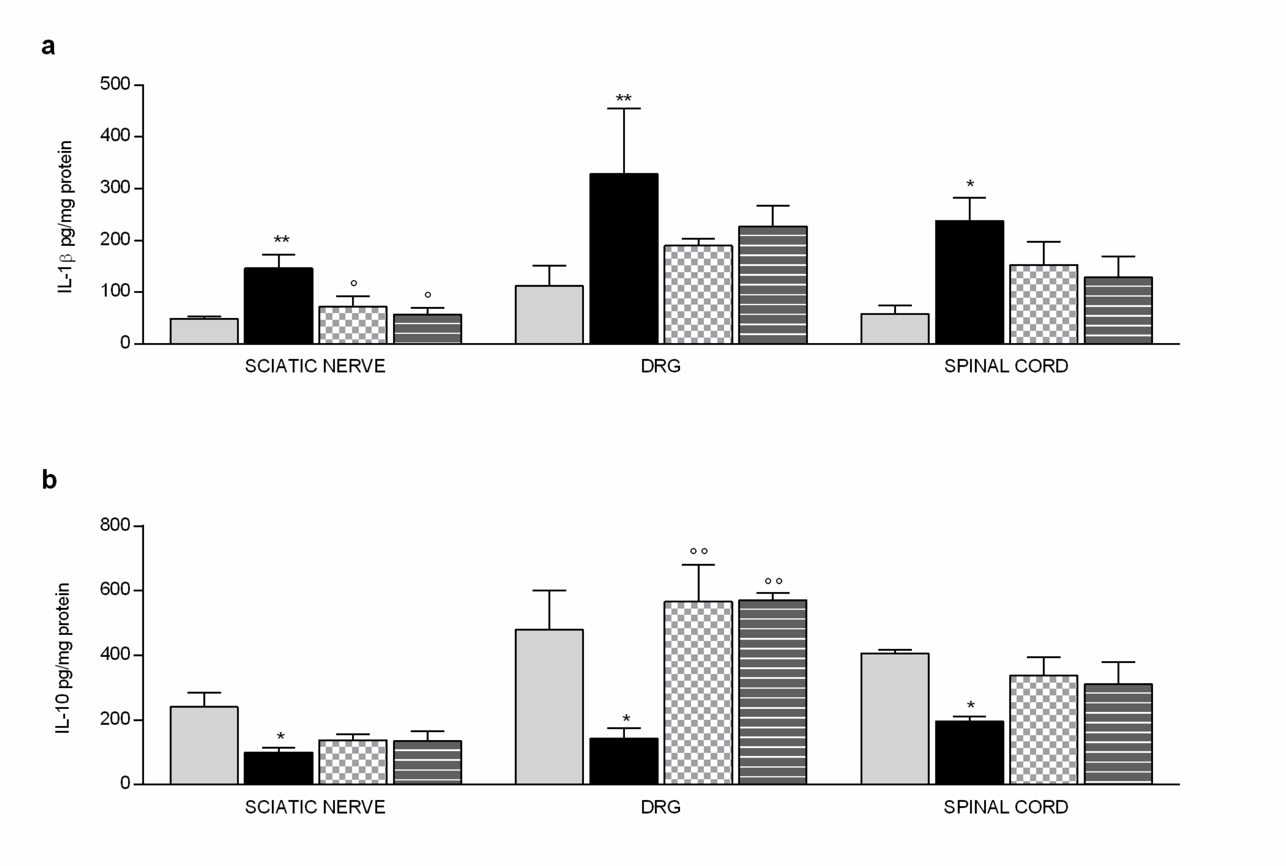

Supplementary Fig. S1**. Effect of hASC and hASC-CM on IL-1β and IL-10 in sciatic nerves, DRG and spinal cord of STZ mice.** IL-1β (**a**) and IL-10 (**b**) protein content in nervous tissues was evaluated by ELISA and reported as pg cytokine/mg total protein. Cytokines were evaluated in sciatic nerve, DRG and spinal cord 3 hours after hASC or hASC-CM treatment, two weeks after STZ. Data represent mean ± SEM of 6 mice per group. One-way ANOVA was used for statistical evaluation, followed by Bonferroni's post hoc test for multiple comparisons.*$p<0.05$, **$p<0.01$ vs CTR; °$p<0.05$, °°$p<0.01$ vs STZ

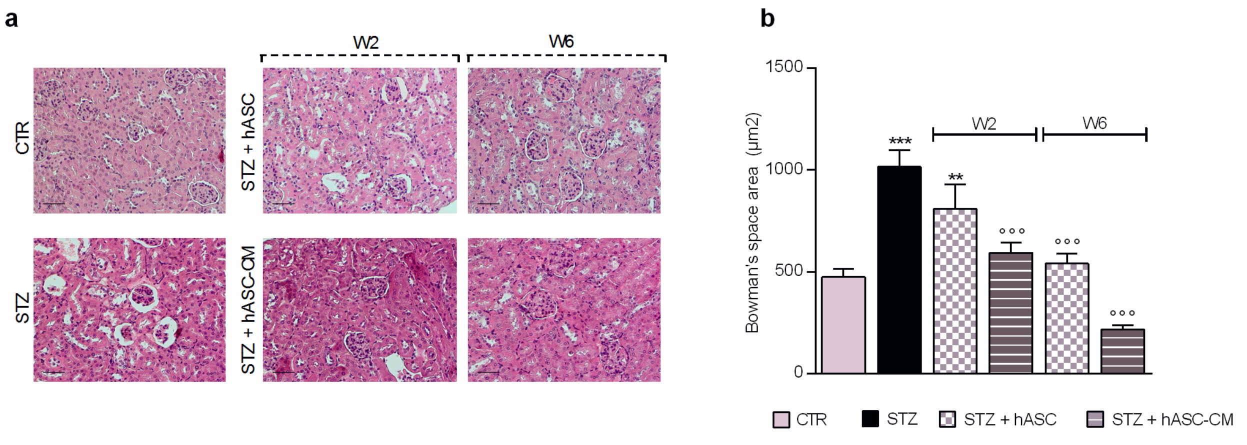

Supplementary Fig. S2**. Effect of hASC and hASC-CM on glomerular damage in STZ mice**

a: Hematoxylin/eosin staining of kidney sections (magnification: 200X) from CTR, STZ and STZ treated with hASC or hASC-CM, 2 weeks (W2) or 6 weeks (W6) after diabetes induction. **b**: quantification of Bowman's space area for all the animal groups. More than 30 glomeruli were measured for each group. Data were analyzed and compared by One-way ANOVA and by a Bonferroni's multiple comparison test and are expressed as mean ± SEM. **p< 0.01, ***p< 0.001 vs CTR; °°° p< 0.001 vs STZ.
